# Supplementary material for: Identification of photoperiod-induced specific miRNAs in the adrenal glands of Sunite sheep (Ovis aries)
Source: Front Vet Sci. 2022 Jul 22;9:888207. doi: 10.3389/fvets.2022.888207 (PMC9354845; doi:10.3389/fvets.2022.888207)
Supplement: Supplementary file 1 [file Data_Sheet_1.ZIP › Supplementary file/miRDeep2 file.zip!/miRDeep2 file/Know_Structure/LP42A3/oar-mir-134.pdf]

miRBase precursor : oar-mir-134
Total read count : 89
oar-miR-134-5p read count : 81
oar-miR-134-3p read count : 8
remaining reads : 0

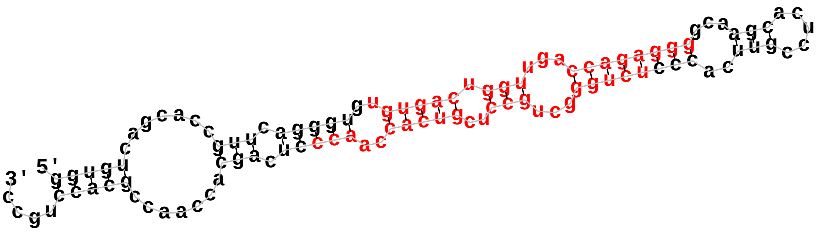

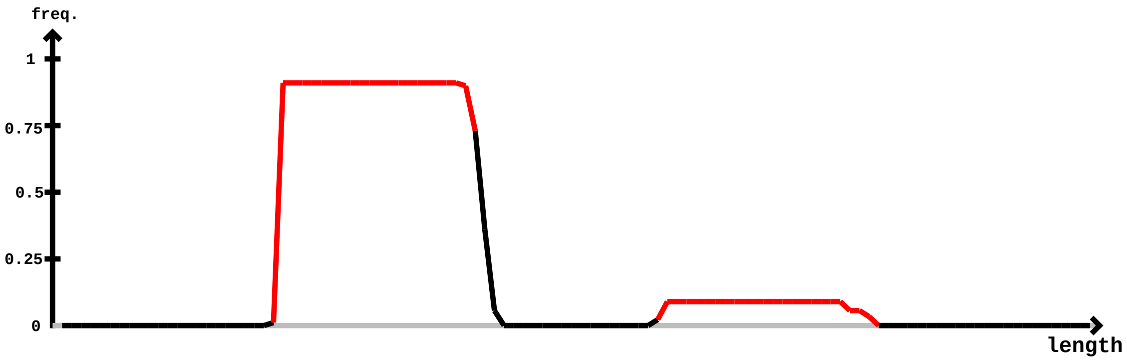


```
                                                                   oar-miR-134-3p
                        oar-miR-134-5p
5'- ggugucagcaccguucagggugugugacugguugaccagagggcaagcacuccguucacccucugggcugccucgucaccaacccucagcaccaaccgcaccugcc   -3'    exp
    (((((.......(((.(((((..(((((.(((...((((((((...(((.....)))..))))))))...)))..)))))..))))).))).......))))).... reads      mm         sample
    .....................gugugacugguugaccagaggg................................................................ 1          0          seq
    ......................ugugacugguugaccagag.................................................................. 1          0          seq
    ......................ugugacugguugaccagagg................................................................. 15         0          seq
    ......................ugugacugguugaccagaggg................................................................ 32         0          seq
    ......................ugugacugguugaccagagggg............................................................... 27         0          seq
    ......................ugugacugguugaccagaggggc.............................................................. 5          0          seq
    .............................................................cucugggcugccucgucacc.......................... 1          0          seq
    .............................................................cucugggcugccucgucaccaa........................ 1          0          seq
    ..............................................................ucugggcugccucgucacc.......................... 2          0          seq
    ..............................................................ucugggcugccucgucaccaa........................ 1          0          seq
    ..............................................................ucugggcugccucgucaccaac....................... 3          0          seq
```
